# Detection of atypical genes in virus families using a one-class SVM

**DOI:** 10.1186/1471-2164-15-913

**Published:** 2014-10-20

**Authors:** Saskia Metzler, Olga V Kalinina

**Affiliations:** Department for Computational Biology and Applied Algorithmics, Max Planck Institute for Informatics, Campus E1 4, 66123 Saarbrücken, Germany

**Keywords:** Horizontal gene transfer, Viral evolution, Machine learning, SVM

## Abstract

**Background:**

The diversity of viruses, the absence of universally common genes in them, and their ability to act as carriers of genetic material make assessment of evolutionary paths of viral genes very difficult. One important factor contributing to this complexity is horizontal gene transfer.

**Results:**

We explore the possibility for the systematic identification of atypical genes within virus families, including viruses whose genome is not encoded by a double-stranded DNA. Our method is based on gene statistical features that differ in genes that were subject of recent horizontal gene transfer from those of the genome in which they are observed. We employ a one-class SVM approach to detect atypical genes within a virus family basing of their statistical signatures and without explicit knowledge of the source species. The simplicity of the statistical features used makes the method applicable to various viruses irrespective of their genome size or type.

**Conclusions:**

On simulated data, the method can robustly identify alien genes irrespective of the coding nucleic acid found in a virus. It also compares well to results obtained in related studies for double-stranded DNA viruses. Its value in practice is confirmed by the identification of isolated examples of horizontal gene transfer events that have already been described in the literature. A Python package implementing the method and the results for the analyzed virus families are available at http://svm-agp.bioinf.mpi-inf.mpg.de.

## Background

Viruses interact extensively with the host cells they infect, and thus are believed to play a major role in the evolution of life [1]. In the process of virus-host interaction, viral genes might be left behind and incorporated into the host genome, or host genes might be taken up by viruses and become integrated into the viral genome. In this work, we devise a model to systematically predict which viral genes do not originate from the species in which they were observed. The method is targeted to the identification of genes that presumably have been integrated into virus genomes only recently.

Horizontal gene transfer (HGT) amounts to the exchange of genetic material between organisms other than by reproduction and typically across species boundaries. Instances of HGT have been observed in all three domains of life [2] as well as in viruses. For the identification of horizontally transferred genetic material, various methods have been proposed (reviewed in [3]). In general, the methods are based on either phylogenetic analysis or interpretation of compositional features.

Phylogenetic analysis requires assumptions about a comprehensive phylogenetic tree. While cellular organisms have a common ancestor, viruses likely do not originate from a common progenitor [4]. Moreover, viral genomes evolve more quickly than cellular genomes, which very quickly causes genes to diverge beyond any recognizable similarity. Hence, phylogenetic trees can be constructed only for a limited set of closely related viruses; and methods based on phylogeny are applicable to the study of HGT in viruses only to a limited extend. In contrast, methods based on compositional features are applicable. These methods involve the analysis of statistical features and specific genomic signatures, and are more powerful in detection of gene insertion, as opposed to phylogeny-based techniques that can detect gene displacement as well [1].

A genomic signature is a vector of numbers constructed from a DNA or protein sequence. These vectors are similar for all sequences from the same genome (i.e. pervasive) and distinct from vectors corresponding to sequences of other genomes. Species evolve specific signatures in the characteristic evolutionary processes each of them is subjected to in time. Comparison of these patterns enables the identification of horizontally transferred genes as those whose features are atypical for a particular genome. However, only recently acquired genes can be detected because sequences quickly adjust to their new genome environment, a process known as gene amelioration [5].

The statistical analysis of viral genetic data is challenging. These data exhibit high biases due to comparably short genomes and the tendency of certain viruses to display high mutation rates. Yet, there is a number of studies focusing on HGT in viruses.

For cyanobacterial phages and their hosts, it has been shown that whole genes of the photosystem reaction center have been transferred from host to phage [6]. The host-like photosynthesis genes of the cyanophages presumably augment the host photosynthetic machinery during infection and thereby provide a fitness advantage. There is also evidence for the transfer of multiple genes involved in the sphingolipid biosynthesis pathway between the eukaryotic microalga *Emiliania huxleyi* and its large double stranded DNA virus Emiliania huxleyi virus although the direction of the HGT was not identified [7]. The similarity of *polA* genes in both thermophilic viruses and the bacterial phlyla of *Aquificae* and *Apicomplexa* could be attributed to not only one event of gene transfer but to a whole network of transfers of genetic material that occurred during evolution [8]. A study of HGT in the family of *Poxviridae* has revealed that proteins encoded by members of the subfamily *Chordopoxvirinae* exhibit greater similarity to eukaryotic proteins than to proteins of other virus families illustrating the important role of gene capture from the host for virus evolution [9].

A special case is Sputnik virus. This virus infects Acanthamoeba polyphaga mimivirus, one of the largest known viruses. Some of the genes of Sputnik virus apparently originate from *Mimivirus* [10]. Sputnik virus is the first virus discovered to infect another virus [11].

All these studies focus on individual viral species or at most one family and reveal interesting details for them. A number of methods employ machine learning techniques to discover HGT events on a large scale (for a review and comparison, see [12]). None of these methods, however, analyzed viruses specifically as a subject for HGT. Although for Wn-SVM [13] predictions in 106 virus species are reported, the authors concerntrate exclusively on dsDNA viruses with relatively large genomes. In contrast, we aim to provide a method equally applicable for detection of HGT in all known viruses. The method relies on detection of genes whose statistical properties are for the virus it resides in, and thus does not reveal the source of the gene transfer. Recent metagenomic studies indicate a large and partly underappreciated diversity of viral species [14–16], so we speculate that in many cases the source organism may still be undiscovered. The mentioned anecdotal cases are not sufficient to form a gold standard data set of viral genes acquired via HGT. So the validation of the model is particularly demanding. We evaluate the algorithm on simulated data sets as well as on real data sets, and we relate selected findings to available literature.

The identification of potentially horizontally transferred genes resembles an outlier detection problem. We hypothesize that most genes of a virus family are inherent and only few entered the family by means of horizontal transfer. Furthermore, the family-inherent genes share characteristics which the foreign genes do not have. Genetic sequences inherent to a genome exhibit specific common statistical features, whereas genes recently horizontally transferred into the genome show the specific statistical features of their original genome. Thus, representations of genes in the form of signatures allow for distinction of within-family and out-of-family genes.

However, reliable statistics of viruses are hard to obtain due to fast evolutionary rates and short genomes. Representations might be affected by high fluctuations and potentially exhibit large statistical errors. In particular for high-dimensional representations, biases can be extremely high. Options to overcome this are either to work with simple representations or to reduce biases. Representations obtained from either option are evaluated as inputs for the devised algorithm.

The central element of the algorithm is a one-class support vector machine (SVM). In contrast to a regular SVM, a one-class SVM does not require labeled data and hence is particularly suited for the task of outlier detection in virus families where labels are unavailable. It learns a decision function for outlier detection. New data provided to the algorithm are classified as similar or different to the data before seen [17, 18].

## Methods

### One-class SVM

Given all genes of a virus family, a one-class SVM is employed to obtain a ranking of genes from most atypical to most typical. It predicts whether new data is like the data on which it has been trained. Similarly to a regular SVM, the one-class SVM maps data into a feature space by means of a kernel function. In this space, a maximal separating hyperplane is set up such that it separates the data from the origin – manifesting a decision boundary – and maximizes the margin. For a new point, the class membership is determined by evaluating on which side of the hyperplane it falls.

The solution heavily depends on the parameter *ν* that corresponds to the fraction of data points expected to be on the other side of the decision boundary with respect to the origin. Therefore, the one-class SVM is also referred to as *ν*-SVM. The position of the decision boundary is influenced by the choice this parameter. For the prediction of atypical genes, we employ a one-class SVM with a Gaussian kernel. This kernel, particularly suited for real-valued feature sets, requires the proper choice of a second parameter *γ* controlling the variance of the radial basis function. The selection of values for the parameters *ν* and *γ* is demanding as no information on the expected number of outliers is available beforehand. Because of the unsupervised nature of the problem, typical parameter selection procedures, such as cross validation, are infeasible.

We note that although in its original formulation, the one-class SVM is an approach to binary classification, i.e. differentiation between in-class and out-of-class data, it can well be used to rank the data. To obtain a ranking, the signed distances of each data point from the decision boundary are evaluated. Points outside the boundary have distances with a negative sign. For points within the boundary, the distance to the boundary is positive. Hence a ranking from most atypical to most typical is obtained by enumerating the genes with respect to their distance to the decision boundary in ascending order. Because the exact location of the decision boundary is of minor importance for this task and there is no notion of the expected number of outliers, we select parameters such that a ranking is robustly determined. As discussed in detail in this section, the ranking is considered robust if it remains stable under slight variations of the parameters.

### Input features

From the genetic sequences of each virus family, various input features are derived to serve as a input to the one-class SVM. Virus family refers to the taxonomic rank as assigned by the International Committee on Taxonomy of Viruses (ICTV) [19]. These features include

- Oligonucleotide frequencies: The alphabet *Σ*={*A*,*C*,*G*,*T*} has |*Σ*|^*k*^ words of length *k*. The oligonucleotide signature is a |*Σ*|^*k*^-dimensional vector of word frequencies. We employ values of 1, 2, 3, and 4 for *k* to derive feature sets. We do not use oligonucleotide frequencies with *k*>4, because for some families, there is not enough statistics for such high-dimensional feature spaces.
- Codon usage: As features, we use a vector of the frequencies of each codon within a genetic sequence. Relative codon usage is a vector of codon frequencies relative to the occurrence of synonymous codons.
- Amino acid frequencies: The 20-dimensional vector of amino acid frequencies is obtained from a protein sequence from the alphabet *Σ*^′^={*A*,*R*,*N*,*D*,*C*,*E*,*Q*,*G*,*H*,*I*,*L*,*K*,*M*,*F*,*P*,*S*,*T*,*W*,*Y*,*V*}. This vector can equally be derived from the respective genetic sequence by translation of the codons into amino acids.
- Position-based nucleotide frequencies: Given a genetic sequence, the frequencies of nucleotides in the three codon positions yield a 12-dimensional feature vector.
- GC content: The GC content is the proportion of C and G in a sequence from the alphabet *Σ*.

We compare the performance of the algorithm with these nine different input feature sets. With respect to the amount of information, the GC content is the most primitive feature set. This one-dimensional signature is frequently used for the detection of HGT [20, 21] and hence we use it as a base line to assess performance. It is not provided as input to the one-class SVM. Instead, the genes of a virus family are ranked by their GC content and then a ranking is induced by their deviation from the median GC content of the virus family.

In addition to the exploration of different input features, we examined the use of linear regression on the GC content with each of these feature sets in advance of providing them to the one-class SVM. As viruses typically have short genomes and exhibit high mutation rates, this regression step, successfully applied in metagenomics [22], was thought to reduce bias in the data. However, because the data is standardized to zero mean and unit variance before supplied to the SVM, linear regression of any feature set yields the same solution.

### Parameter selection

To choose values for the parameters *ν* and *γ*, we explore a wide range of them. The aim is to achieve stability of the ranking of the data points induced by the signed distances from the decision boundary. A stable result is not necessarily correct but more trustworthy than a result that sensitively varies under small disturbances. The parameter *ν* takes 1, 000 equidistant values in the interval (0,1]. The parameter *γ* is explored at 10^−6^, 10^−5^, 10^−4^, 10^−3^, 0.01, 0.1, 1, 10, 100, and (2*D*)^−1^, where *D* is the dimensionality of the respective feature space. The latter value for *γ* is frequently used as a rule-of-thumb value for SVMs with Gaussian kernel. The similarity of the ranked lists, obtained for successive values of *ν*, is measured by Spearman’s rank correlation *ρ* [23].

### Data sets

We consider all viral genes from ENA, the European Nucleotide Archive [24], downloaded in July 2013. These data are clustered using CD-HIT [25] with a sequence identity level of 95*%*. Representatives of each cluster, grouped by their virus family, yield the data sets. The data sets form the basis to derive feature sets presented as input to the prediction algorithm.

From the initial data sets, simulated data sets for each virus family are created using all genes of that family and adding portion of artificial outliers chosen uniformly at random from all viral genes not from this family such that 5*%* of the genes in the artificially amended family are outliers. These data with simulated outliers allows for the use of measures of binary classification performance during the evaluation. However it should be pointed out that the actual outliers remain unrecognized in the data and compete with the artificially added outliers. We did not perform any comparison with proven events of HGT, since no collection of such events exists for viruses.

### Measure of performance

Area under curve (AUC) scores are used to evaluate the performance in the simulated setting. AUC is defined as the area under a receiver operator characteristics (ROC) curve. The ROC curve for binary classification is a plot of the true positive rate as a function of the false positive rate when different cutoffs for the number of predicted atypical genes are considered. For the simulated data sets, the upper 5*%* of the list are considered as predicted atypical genes.

The performance of the prediction method was also assessed by measuring the AUC scores when the labels are inferred from BLAST searches [26] for each gene in the result. Genes are considered atypical if they have at least one bi-directional BLAST hit outside the viral family (i.e. the hit is significant when searching with the sequence of the gene from the viral family and from outside the family), and the sequences of the two genes are similar over 80*%* of the length of the family gene. As this labeling can at most reveal a trend, additional literature search is conducted for a number of genes to strengthen the analysis. The BLAST search is executed on protein level using BLAST version 2.2.25 with default parameters querying the UniProt database [27].

## Results and discussion

The expected result of the one-class SVM, given a feature set and parameters *ν* and *γ*, is a ranked list, sorted from the most atypical gene to the most typical gene in the respective virus family. Before analyzing the performance, initial focus is on the selection of appropriate parameters *ν* and *γ*.

### Robustness of the prediction

**Figure 1:**
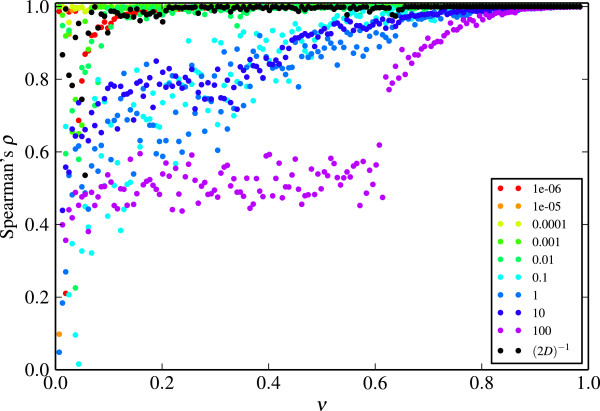
**Ranking stability under varying** ***ν*** **for different values of** ***γ*** **.** Correlation between prediction results under varying *ν* for different values of *γ* (corresponding to the different colors). The prediction results originate from *Partitiviridae* using codon usage estimates as feature set. Every point corresponds to the correlation between the prediction result for one value of *ν* and the prediction result for the next value of *ν*, averaged with the correlation between the result for current value of *ν* and the previous. The step size between subsequent values of *ν* is 0.001.

The assessment of ranking stability is illustrated for *Partitiviridae* and a feature set based on codon usage estimates (Figure 1). Each point depicts the average Spearman’s rank correlation coefficient over the prediction result with the neighboring values of *ν*. A value of 1 indicates that the ranking obtained with the current value of *ν* is identical to the ranking resulting from the preceding value of *ν* and the subsequent value of *ν*.

For *ν*>0.8, the ranking remains invariant under changes of *ν* irrespective of the choice of *γ*. The smaller *ν* is, the smaller the observed correlation between neighboring parameter sets. Additionally, the stability depends on *γ*. For small values of *γ*, the stability is generally better than for large values of *γ*. The prediction result obtained with large values of *γ* can be very sensitive to small deviations in *ν*. For some feature sets (not displayed), if the value of *γ* is large, there is not even a trend towards increasing stability when *ν* is large.

For all parameter sets and for all virus families, we observe that *γ*=(2*D*)^−1^ yields a high stability over a large range of *ν*. This finding confirms that this rule of thumb for the selection of *γ* is a reasonable choice. Thus, we refrain from conducting the subsequent evaluation for multiple values of *γ* and fix it to *γ*=(2*D*)^−1^.

The parameter *ν* impacts the proportion of outliers we expect to observe in the data. This proportion however is unknown. The ranking stability analysis indicates that assuming the proportion of outliers to be very low might yield an unstable prediction result. Because we do not rely on the binary labels from the one-class SVM result but rather on the signed distances to the decision boundary, we can fix *ν* such that the ranking is stable irrespective of assumptions on the actual number of alien genes.

To evaluate the prediction algorithm for the different sets of input features and different SVM parameters we establish a measure of success. The prediction of atypical genes in a viral family is considered successful if the AUC score exceeds 0.9 and semi-successful if it only exceeds 0.8 in the simulated data setting. The prediction for a family is considered successful if success is reported for at least one combination of feature set and parameters.

**Figure 2:**
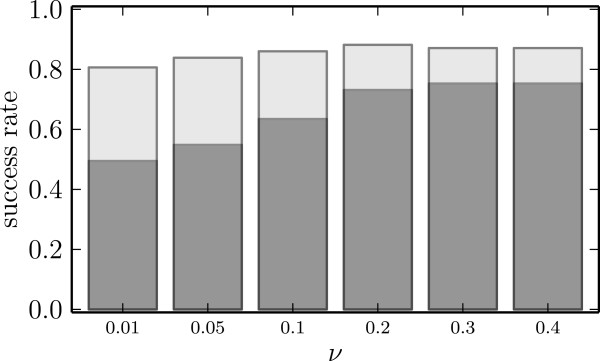
**Success rates of the one-class SVM for different values of** ***ν*** **.** The proportion of successfully predicted families is depicted in darkgray, the proportion of semi-successes in lightgray.

Values of *ν* smaller than 0.1 yield less successes than larger *ν* (Figure 2). The semi-success rates are constantly around 80*%*. Among the larger *ν*s, there is no preference for a specific value. We fix *ν*=0.2 for the subsequent analysis.

### Prediction quality on simulated data

The assessment of performance on simulated data shows the quality of the established model.

From the ENA database [24] we obtain 93 virus families with more than three genes recorded. For 83 of them, our method is capable of identifying the alien genes with AUC score larger than 0.8 in at least one set of features (Figure 2). For 70 of the families the AUC scores are larger than 0.9. The number of genes in the families ranges from less than 100 to more than 10, 000 for *Phycodnaviridae* and *Flaviviridae*. The family of *Retroviridae* is too large to be processed as a whole and is split up taking a lower level of taxonomy, the level of genus. On this level, the genus *Lentivirus* still comprises more than 100, 000 genes from which we sample only 25, 000 uniformly at random.

The large deviations in the number of genes per family are the result of a bias in research intensity. The family of *Flaviviridae* for example comprises viruses such as Dengue virus, Yellow fever virus, Hepatitis C virus, and Swine fever virus. These viruses are infectious to humans or domestic animals and are therefore of particular interest. The genus *Lentivirus* includes HIV which is subject of intensive research as it causes the acquired immunodeficiency syndrome (AIDS). *Phycodnaviridae* infect algae and are probably so numerous in the database because of metagenomic studies of water samples.

**Figure 3:**
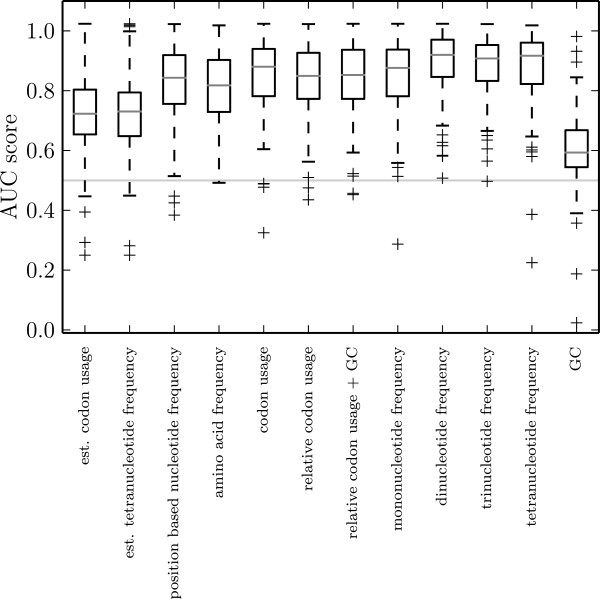
**Boxplot of AUC scores on simulated data for different feature sets.** Gray bars indicate the median score observed in all families and 50*%* of all scores fall within the range indicated by the black boxes. The horizontal line at 0.5 highlights the score expected for a random result.

Comparing AUC scores obtained in all families for different feature sets we find indications of very good performance. In particular in comparison with the primitive prediction method based on GC content, the quality of the approach becomes evident: The median AUC scores are 0.59 for that method and between 0.82 and 0.92 for the more sophisticated feature sets. Median scores above 0.9 are observed only for oligonucleotide frequencies (Figure 3). Generally, the oligonucleotide frequencies of length *k*≥2 appear to be the most preferable feature sets, tetranucleotide frequencies being the best performing.

For completeness, we display also sample results obtained using linear regression for the derivation of the feature sets (first two columns of Figure 3). The median score with these feature sets is 0.72, which is inferior to the performance of feature sets without regression. The difference in performance explains by the fact that there are *de facto* only two distinct dimensions as explained in the previous section.

### Comparison of the prediction results with the naïve labeling using BLAST

We have compared out prediction results with potential HGT genes identified by BLAST [26]. The assumption behind this is that if a HGT event is predicted correctly, we may find a related gene in an organism outside the immediate viral family. Thus, we label a gene as atypical according to BLAST if there is at least one bi-directional BLAST hit outside the virus family under consideration with the similarity observed over at least 80*%* of the query gene length. This labeling implies many shortcomings. Besides biases from the fact that different organisms are studied with different intensity, it does not differentiate between hits that can occur due to gene transfer from the particular family to another and those that occur due to gene transfer into the family or that may be a signature of a common ancestry. While with this naïve labeling, all three cases are classified atypical, the prediction algorithm is supposed to identify only the second case. The observation of concordance between the prediction result and the labeling derived from the BLAST searches, despite these shortcomings, provides support, yet no proof, for the established methodology. On the contrary, inconsistency between the labeling and the prediction result does not imply failure of the prediction algorithm. In any case, the most valuable support, if at all, has to be provided by extensive literature search.

**Figure 4:**
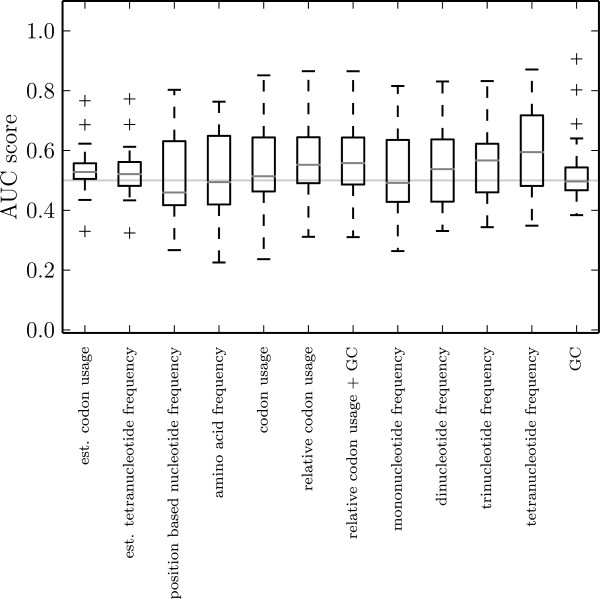
**Boxplot of AUC scores between the one-class SVM prediction results and the BLAST search results on different feature sets.** Only 22 families are taken into account. The horizontal line at 0.5 highlights the score expected in case of a random result.

Despite their effectiveness, BLAST searches become infeasible for large virus families. Therefore, we restrict this analysis to 22 families. Considering the distributions of scores from all these families together yields no indication of success for any of the feature sets. All median scores are close to 0.5, the highest being 0.59 for the tetranucleotide frequency feature set (Figure 4). Codon usage-based feature sets produce second-largest AUC scores of 0.55. Considering this together with the performance results for the simulated data, we conclude that codon usage-based tetranucleotide frequency feature sets produce most reliable results. Overall, we do not observe any significant correlation between the predictions of our algorithm and the labeling inferred from BLAST. However, for some families, we observe the prediction result and the labeling inferred from BLAST searches to be partially in conjunction with each other, as manifested by AUC scores much higher than 0.5. A few such examples are discussed below.

#### Orthomyxoviridae

Many feature sets perform well for this family, as demonstrated by the average AUC score of 0.79. Interestingly, the GC content privedes very good agreement with BLAST-based annotation, rendering an AUC score of 0.91.

The most prominent members of *Orthomyxoviridae* are influenza viruses. They cause infectious diseases in mammals and birds spreading in seasonal epidemics. *Orthomyxoviridae* are viruses with single stranded anti-sense RNA which implies that the nucleotide frequencies might deviate from Chargaff’s rule [28, 29]. The atypical genes identified by both the labeling inferred from BLAST searches and the one-class SVM exhibit nucleotide frequencies in accordance with Chargaff’s rules while a large proportion of genes, mostly from *Influenzavirus A*, show opposing behavior in the frequencies of adenine and thymine.

Influenzavirus A is the subject of intensive study, and its genes constitute 95*%* of the genes of the *Orthomyxoviridae* family in our data. For the one-class SVM approach, it is possible that this imbalance influences the identification of atypical genes. The BLAST algorithm however is independent of statistical signatures of viral families and yields a similar result: Atypical genes are found among genes not from Influenzavirus A and typically have high GC content. Of a total of 15 atypical genes identified by the BLAST search, none are from Influenzavirus A. The high evolutionary rate in the Influenza A virus attributed to positive selection through the human immune system [30] and a very narrow specialized set of its proteins can be the reason why the horizontally transferred genes are not accommodated by this virus.

Support for our method is provided by the identification of the Araguari virus partial glycoprotein (ENA accession ABB55449.1), Dhori virus partial glycoprotein (CAA66028.1), Batken virus partial glycoprotein (CAA66030.1), and several partial glycoproteins from Thogoto virus strains (AAL31456.1, AAL31461.1, AAL31462.1) as atypical genes. These genes occur within the top 100 of 3571 in the ranked list result when using the tetranucleotide frequencies feature set. All these viruses belong to the *Thogotovirus* genus. The Thogoto virus glycoprotein has been found to have resemblances to the baculoviral glycoprotein GP64 indicating a possibility of gene transfer [31].

#### Circoviridae

In *Circoviridae* we observe the AUC score to reach 0.72 for the tetranucleotide frequency feature set. *Circoviridae* have a circular single-stranded DNA genome. They are broadly distributed among vertebrates without causing illnesses for most organisms. The BLAST hits of the top ranked genes refer to a variety of species: Chicken anemia virus partial protein VP2 (ACT66124.1) is found to exhibit similarity to the RmuC-domain protein (CDB07518.1) from *Odoribacter splanchnicus*; and a protein of unknown function from Duck circovirus (AAZ07882.1) is similar to serine/threonine protein kinase related protein (AHC15994.1) from *Spirochaeta sp*. Furthermore, a number of replication-associated proteins, including Rep (AEL28813.1) from Bat circovirus ZS/Yunnan-China/2009, the putative Rep (AFH02742.1) from Circoviridae batCV-SC703 are similar to a Rep-like protein (AAR83499.1) of Canarypox virus, a dsDNA virus. The multiple cases of similarity between the Rep protein to proteins from unrelated viruses hint to a complex history of this protein, which has been corroborated by previous research: Meehan *et al.* hypothesize that Rep has originated through recombination, combining gene segments from unrelated viruses [32]; and Gibbs *et al.* identified Rep-like genes by means of a database search and speculate that these genes originated by multiple interspecies recombinations as they are represented in viral, plasmid, bacterial, and parasitic protozoan genomes [33].

#### Flaviviridae

For *Flaviviridae*, we observe an AUC score of 0.70 for the position-based nucleotide frequencies feature set. *Flaviviridae* have a single-stranded sense RNA genome and are often spread through ticks and mosquitoes. They are subject of intense studies as they are infectious to humans and domestic animals. The majority of *Flaviviridae* genes in ENA stems from Hepatitis C virus. A sub-population of these genes, like Influenzavirus A genes in *Orthomyxoviridae*, exhibits strong deviations from Chargaff’s rule in their nucleotide composition. The prediction algorithm as well as the labeling based on the BLAST search identify atypical genes from Wang Thong virus (AAS16519.1) and Culex flavivirus (BAH83691.1). The corresponding non-viral genes are found in Asian tiger mosquito and Yellowfever mosquito, likely hosts of these viruses. Thus this represents most probably the inverse event of horizontal gene transfer from virus into its host.

#### Hepadnaviridae

For *Hepadnaviridae*, the AUC scores reach 0.86 for relative codon usage and tetranucleotide frequencies feature sets, indicating good correlation between the labels inferred from BLAST searches and the prediction result. *Hepadnaviridae* have a reversely transcribed double-stranded DNA genome that get integrated into the host genome for transcription during their replication process. *Hepadnaviridae* infect mammals and birds. All the BLAST hits outside *Hepadnaviridae* can be explained by the integration of the virus genome into the host. They stem from a sequence analysis of hepatocellular carcinoma in hepatitis B infected woodchuck [34] and from a study on budgerigar genomes being infiltrated by hepadnaviruses millions of years ago [35]. The BLAST search in this case provides no information on the validity of the SVM based prediction.

Given the average AUC scores around 0.5 for all examined families (Figure 4), the question rises whether the SVM prediction and the BLAST search actually have a reasonable overlap that allows for conclusions. At least for *Hepadnaviridae*, and potentially also for other retro-transcribing viruses, the labeling inferred from BLAST is not informative and hence allows for no statement about the prediction performance of the SVM.

#### Siphoviridae

For some virus families, AUC scores of at most 0.5 indicate poor prediction results. *Siphoviridae* is one example where scores below the performance of a random classification are observed. The reason for these scores is that almost every gene from *Siphoviridae* yields BLAST hits outside the family and thus, to attain a good score, the few genes with no hits outside would need to be at the very end of the ranked result and not somewhere in the middle. Our method however is tuned for the identification of a minority of atypical genes and not a minority of typical genes. The test with the BLAST search appears to be not applicable in this case.

*Siphoviridae* are bacteriophages and can shuttle genes between bacterial species. This mechanism, known as transduction, is a common mechanism of horizontal gene transfer among bacteria. Therefore, it is not surprising that many genes from *Siphoviridae* have bacterial counterparts which are detected by the BLAST search.

This example shows that the prediction quality on real data is hard to assess. Good or bad AUC scores in comparison with BLAST labeling are a sign neither of failure nor of success of the prediction algorithm. They only associate two labeling procedures and neither can be validated. However, if there were a perfect validation strategy, it would already solve the problem.

### Comparison to other available methods

Machine learning techniques have been employed for the identification of horizontal gene transfer events before: specifically, composition-based statistics have been used by Tsirigos and Rigoutsos [13] and Dufraigne *et al.* [36]. In both studies, statistics were collected from segments identified by a sliding window over a genome of a single species. Either tetramers in conjunction with distance-based prediction [36], or complex compositional feature vectors and one-class SVMs [13] can be used. Our approach bears similarity to both of these methods, but works on the level of complete genes and viral families. The details of the implementation of the SVM also differ: while we fix parameters beforehand and report the ranking induced by the distances of each gene to the decision hyperplane, Tsirigos and Rigoutsos estimate the parameter *ν* such that, in an artificial setting, the ratio of recovered artificially inserted genes is maximized. As this kind of selection is infeasible in a real setting, regions are reported as a candidate for gene transfer only if they were marked as atypical at a number of tested values of *ν*. For the sake of comparison, we have modified the algorithm to use single species genomes and a sliding window of the size coinciding with the respective study.

**Figure 5:**
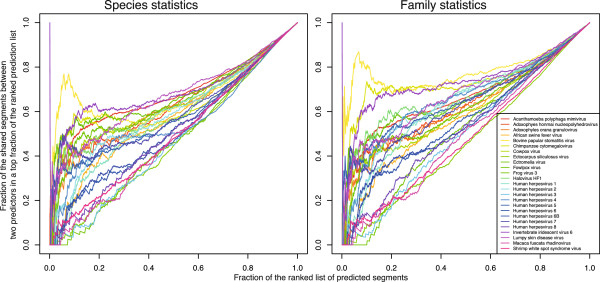
**Comparison with** [13]**.** Each curve on the plot represents the fraction of the shared segments in a top fraction of the lists generated by our algorithm and reported in [13]. The tetranucleotide statistics in our method were calculated using a single species sequence (left) and all genes from the corresponding family (right).

**Figure 6:**
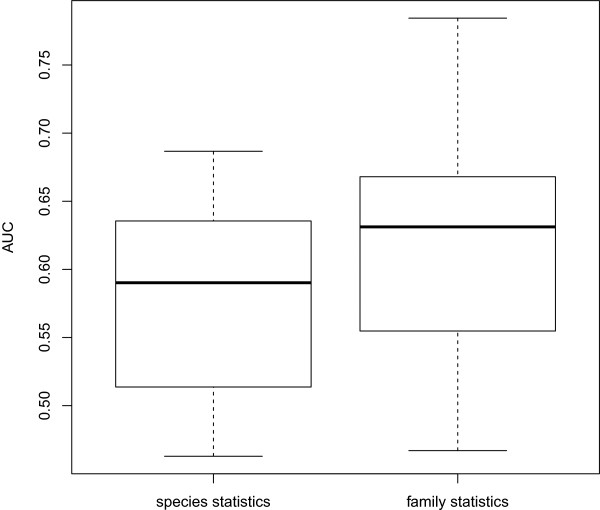
**Distribution of AUC scores in comparison with** [13]**.** Boxplot of AUC scores corresponding to the comparison of the results of our method and that of [13]. The tetranucleotide statistics in our method were calculated using a single species sequence (left) and all genes from the corresponding family (right).

Tsirigos and Rigoutsos [13] report the prediction of regions of atypical composition ranked from most to least atypical in 106 viruses from the families of *Asfaviridae*, *Baculoviridae*, *Herpesviridae*, *Iridovoridae*, *Nimaviridae*, *Nudiviridae*, *Phycodnaviridae*, and *Poxviridae*, and unclassified dsDNA viruses. We selected a representative set of 25 viral strains, created a set of segments using the sliding window approach, and ranked them using (*i*) the compositional statistics calculated for the same set of segments, and (*ii*) using statistics for the whole family this virus belongs to (only tetranucleotide frequencies were used, since they were found to perform best in our previous experiments). The comparison with the ranking from [13] shows overall good agreement of the results, although it varies largely among the strains (Figure 5). The curves represent the fraction of the shared segments in a top fraction of the two lists: one from [13], and the other calculated with our method. Interestingly, the usage of family-based statistics appears to compensate for a more simplistic sequence representation (we use only tetramers, whereas Tsirigos and Rigoutsos [13] use *k*-mers of orders up to 8 with wildcards for certain positions), as reflected by AUCs that are significantly higher in this case (Figure 6, p-value 0.09 in one-sided Wilcoxon test). This can make our method potentially better suited for species with small genome size and skewed nucleotide composition, e.g. RNA viruses or retroviruses.

Of the 25 representative viruses, the best agreement with the results of [13] is observed for Bovine papular stomatitis virus (AUC = 0.78), Chimpanzee cytomegalovirus, and Invertebrate iridescent virus 6 (AUC = 0.74 in both cases). It is plausible that Bovine papular stomatitis virus harbors genes that were horizontally transferred to it from its host [37]. The possibility of gene transfer from the host into *Iridoviridae* (the family containing Invertebrate iridescent virus 6) has also been discussed in the literature [38]. We do not have specific information for Chimpanzee cytomegalovirus, but for Human cytomegalovirus, four genes are pointed out as GPCR homologs and thus having potentially eukaryotic origin: UL33, UL78, US12 and US21 [13]. In all of them except US21, we also identify atypical segments with a rank within top 20*%* using family-based statistics.

**Figure 7:**
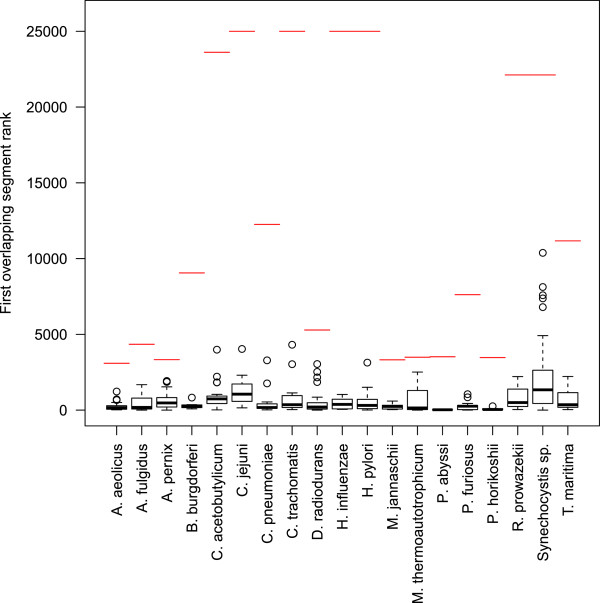
**Distribution of HGT segment ranks in comparison with** [36]**.** Boxplot of ranks of the first overlapping segment with those identified as horizontally transferred by [36]. Red lines indicate the total number of the considered segments.

In another study, 22 bacterial genomes were analyzed to predict the events of HGT using tetranucleotide statistics [36], which makes it also similar to our method. Again, we mimicked this approaches by considering segments of genomic sequence of the corresponding species, and using all genome assemblies from NCBI for each species (instead of viral families) as the source for the tetranucleotide frequency statistics. For 19 of the 22 species we were able to obtain results within reasonable time, and calculated the rank of the first segment ovelapping with a HGT segment reported by [36] (Figure 7). In all cases except *A. pernix* the median of these ranks lies within top 10*%*, which indicates a very good agreement of our methods with that of [36], although our method was not specifically trained to make predictions in Bacteria.

## Conclusions

The presented one-class SVM model convincingly identifies potential examples of HGT in cases when they were artificially added to a family but in many cases does not agree well with labels derived by BLAST. Given statistics about the composition of a viral family, the method robustly identifies the genes that are most atypical for the family. The model can provide no guarantee for the identified genes to be horizontally transferred. Its prediction is based solely on statistics using no external information to attain additional evidence for or against the result. The interpretation of the result remains to be derived manually. Compared to phylogenetic analysis for HGT indetification, out method presents the following advantages: (*i*) it can identify HGT events without any assumptions about the source species (which might have been not discovered yet); and (*ii*) it can operate with much larger sets of genes than even the simplest sequence comparison.

We have specifically tested our algorithm only on real virus sequence data. Unlike artificial data, these data have shortcomings. Aside from issues of correctness and completeness of the gene records, statistical biases are what makes prediction very difficult. While for some viruses there are very few genes in the database, others are the subject of intensive study and thousands of gene records exist. For a virus family of which one member is highly over-represented, the prediction algorithm will regard the genes of this member as typical for the whole family. This bias could be eliminated by sampling down the number of genes per species to that of the species with least genes in the family. The downside of this procedure is a possible loss of information if the that number is very low.

Due to the use of real data, not only the assessment of performance in the real setting is difficult but also the simulated data sets are affected by shortcomings in the data as they are constructed from these data. The simulated atypical genes added to each family are taken from the set of all other viral genes. They are distributed uniformly with respect to the total number of genes other than from the family of interest. Hence, it is biased towards well studied families with many gene records in the database. For this reason, it is possible that the observed performance for families with properties similar to large families is underestimated while the performance established for families with properties very different from the characteristics of large families is overly optimistic. It is also evident that we cannot expect to observe perfect prediction performance on the simulated data as the true unlabeled outliers remain in the data sets and should be identified by the prediction algorithm just like the artificial atypical genes.

For the evaluation of performance on the real data sets, the comparison to the result of BLAST searches is not a perfect strategy. As the one-class SVM approach, the BLAST result is affected by the presence or absence of information as well, yet with different consequences: BLAST does not construct a statistical model for viral families but summarizes which other genes similar to the one queried exist in the UniProt database. If the gene of interest is from a well studied group of related families, very good matches will be found among the genes of common ancestry, occluding matches from other families as we only consider the first 500 hits. In case of gene transfer of a viral gene not into but out of the considered family, this gene is labeled atypical irrespective of its statistical features.

Despite these difficulties, we observe overall reasonable recovery of simulated atypical genes in the data sets and were able to identify genes proposed to be horizontally transferred in the literature. We also note that the presented methodology can be directly applied for the identification of HGT in cellular organisms as well.
